# Clinical outcomes of surgical management for rare types of progressive familial intrahepatic cholestasis: a case series

**DOI:** 10.1186/s40792-022-01365-1

**Published:** 2022-01-13

**Authors:** Kazunori Masahata, Takehisa Ueno, Kazuhiko Bessho, Tasuku Kodama, Ryo Tsukada, Ryuta Saka, Yuko Tazuke, Shuji Miyagawa, Hiroomi Okuyama

**Affiliations:** 1 Department of Pediatric Surgery, Osaka University Graduate School of Medicine, 2-2 Yamadaoka, Suita, Osaka, 565-0871 Japan; 2 Department of Pediatrics, Osaka University Graduate School of Medicine, Osaka, Japan

**Keywords:** Progressive familial intrahepatic cholestasis, Liver transplantation, Cholestasis, Pediatrics

## Abstract

**Background:**

Progressive familial intrahepatic cholestasis (PFIC) is a heterogeneous group of genetic autosomal recessive diseases that cause severe cholestasis, which progresses to cirrhosis and liver failure, in infancy or early childhood. We herein report the clinical outcomes of surgical management in patients with four types of PFIC.

**Case presentation:**

Six patients diagnosed with PFIC who underwent surgical treatment between 1998 and 2020 at our institution were retrospectively assessed. Living-donor liver transplantation (LDLT) was performed in 5 patients with PFIC. The median age at LDLT was 4.8 (range: 1.9–11.4) years. One patient each with familial intrahepatic cholestasis 1 (FIC1) deficiency and bile salt export pump (BSEP) deficiency died after LDLT, and the four remaining patients, one each with deficiency of FIC1, BSEP, multidrug resistance protein 3 (MDR3), and tight junction protein 2 (TJP2), survived. One FIC1 deficiency recipient underwent LDLT secondary to deterioration of liver function, following infectious enteritis. Although he underwent LDLT accompanied by total external biliary diversion, the patient died because of PFIC-related complications. The other patient with FIC1 deficiency had intractable pruritus and underwent partial internal biliary diversion (PIBD) at 9.8 years of age, pruritus largely resolved after PIBD. One BSEP deficiency recipient, who had severe graft damage, experienced recurrence of cholestasis due to the development of antibodies against BSEP after LDLT, and eventually died due to graft failure. The other patient with BSEP deficiency recovered well after LDLT and there was no evidence of posttransplant recurrence of cholestasis. In contrast, recipients with MDR3 or TJP2 deficiency showed good courses and outcomes after LDLT.

**Conclusions:**

Although LDLT was considered an effective treatment for PFIC, the clinical courses and outcomes after LDLT were still inadequate in patients with FIC1 and BSEP deficiency. LDLT accompanied by total biliary diversion may not be as effective for patients with FIC1 deficiency.

## Background

Progressive familial intrahepatic cholestasis (PFIC) is a heterogeneous group of genetic autosomal recessive diseases that cause severe cholestasis, which progresses to cirrhosis and liver failure, in infancy or early childhood [1]. Molecular analysis of patients with this condition has revealed distinct genetic mutations, based on which PFIC is classified into four types. Currently, recognized forms of PFIC (until recently referred to as PFIC types 1, 2, and 3), are characterized by defects in the biliary proteins involved in the formation and flow of bile in the liver. The nomenclature of each entity has changed according to new understanding of these diseases [2]. Familiar intrahepatic cholestasis 1 (FIC1) deficiency is caused by a mutation in the ATPase class 8B member 1 (ATP8B1) gene, which encodes the FIC1 protein. FIC1 is located on the canalicular membrane of the hepatocyte and involved in phospholipid translocation. The protein facilitates movement of phosphatidylserine and phosphatidylethanolamine between the outer and inner leaflet of the plasma membrane of the hepatocyte. Furthermore, it helps to protect the membrane from high bile salt concentration in the canalicular lumen [3]. The FIC1 protein is expressed in the liver, pancreas, small intestine, and kidney, indicating that the deficiency is a systemic disease. The histopathology is characterized by canalicular cholestasis and the absence of true ductular proliferation [4, 5]. Because FIC1 is more highly expressed in the small intestine than in the liver [6], FIC1 deficiency is characterized by cholestatic features and chronic diarrhea [4, 7, 8]. Bile salt export pump (BSEP) deficiency is caused by a mutation in the ATP binding cassette subfamily B member 11 (*ABCB11*) gene that encodes the BSEP protein, only expressed in hepatocytes. The deficiency of BSEP, which plays a role in bile acid excretion, leads to impaired bile salt secretion, causing intrahepatic cholestasis, pruritus, failure to thrive, and progressive liver damage [9, 10]. Multidrug resistance protein 3 (MDR3) deficiency is caused by a mutation in the ATP binding cassette subfamily B member 4 (*ABCB4)* gene that encodes MDR3, which plays a role in phosphatidylcholine excretion. Mutations in the *ABCB4* gene result in absence or low levels of functional MDR3 enzyme leading to decreased level of phospholipids in bile and an abnormality in bile ducts. Consequently, it leads to cholestasis and injury to the biliary epithelium and canaliculi [11, 12]. Tight junction protein 2 (TJP2) deficiency is caused by a mutation in the *TJP2* gene, and defects in tight junctions, leading to severe cholestatic liver disease and extrahepatic manifestations [13–16].

Surgical biliary diversion might be useful for resolving pruritus and has been reported to delay the progressive course of the disease in patients with PFIC [17–19]. Liver transplantation (LT) is considered a curative treatment for patients with PFIC [20–22]. However, some recipients with FIC1 deficiency who undergo LT contributes to the development of refractory diarrhea and graft steatohepatitis [21]. Furthermore, some patients with BSEP deficiency who undergo LT develop recurrent cholestasis due to the development of anti-BSEP antibodies against the liver graft [23–26]. MDR3 and TJP2 deficiency are both extremely rare, and there are only a few reports on the outcomes of LT in pediatric patients with these types [13, 20, 27–31].

We herein report the clinical outcomes of surgical management in patients with four types of PFIC at a single institution.

## Case presentation

### Patients’ characteristics

This study retrospectively assessed six patients with four types of PFIC who underwent surgical treatment at Osaka University Hospital between 1998 and 2020. The demographic characteristics of the patients are shown in Table 1. The diagnosis was confirmed via genetic analysis in four patients (Cases 3, 4, 5, and 6) and via functional analysis of transcription factors in two patients (Cases 1 and 2). Two patients had homozygous mutations, one in the *ABCB4* gene (Case 5) and the other in the *TJP2* gene (Case 6). The other two patients had compound heterozygous mutations in the *ABCB11* gene (Cases 3 and 4). Based on the genetic findings, the patients were diagnosed with four types of PFIC. A mutation was identified in only one allele of the *ATP8B1* gene in Cases 1 and 2, and FIC1 deficiency was diagnosed in both cases based on the clinical presentation and the functional analysis results of the FIC1 protein [32].

**Table 1:** Demographic characteristics of patients with PFIC

Case	PFIC type	Sex	Mutation of gene	Genotype	First symptoms/Age at onset of PFIC	Age at diagnosis (years)	Extrahepatic features
1	FIC1 deficiency	Male	*ATP8B1*	Heterozygous	Jaundice, hepatomegaly/3 months	1.4	Diarrhea, pancreatitis
2	FIC1 deficiency	Male	*ATP8B1*	Heterozygous	Jaundice, hepatomegaly/1 month	0.6	None
3	BSEP deficiency	Male	*ABCB11*	Compound heterozygous	Jaundice, hepatomegaly/1 month	0.5	None
4	BSEP deficiency	Female	*ABCB11*	Compound heterozygous	Jaundice, hepatomegaly/3 months	1	None
5	MDR3 deficiency	Male	*ABCB4*	Homozygous	Abnormal liver function tests, hepatosplenomegaly/2 months	2.1	None
6	TJP2 deficiency	Female	*TJP2*	Homozygous	Jaundice, hepatomegaly/1 month	5	Sensorineural deafness

*PFIC* progressive familial intrahepatic cholestasis, *FIC1* familial intrahepatic cholestasis 1, *BSEP* bile salt export pump, *MDR3* multidrug resistance protein 3, *TJP2* tight junction protein 2, *ATP8B1* ATPase class 8B member 1, *ABCB11* ATP binding cassette subfamily B member 11, *ABCB4* ATP binding cassette subfamily B member 4

### Clinical outcomes of patients with PFIC

#### FIC1 deficiency: cases 1 and 2

**Fig. 1:**
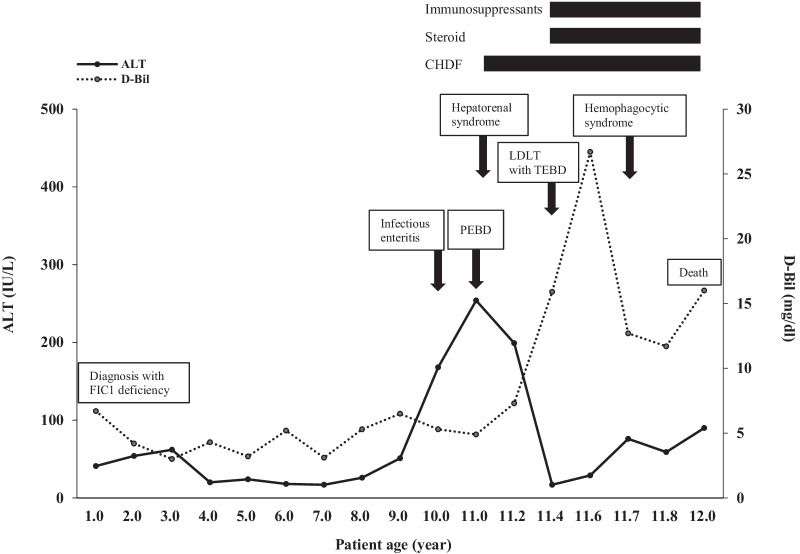
Clinical course of the patient with FIC1 deficiency (case 1). *FIC1* progressive familial intrahepatic cholestasis 1, *ALT* alanine aminotransferase, *D-Bil* direct bilirubin, *CHDF* continuous hemodiafiltration, *PEBD* partial external biliary diversion, *LDLT* living-donor liver transplantation, *TEBD* total external biliary diversion

**Fig. 2:**
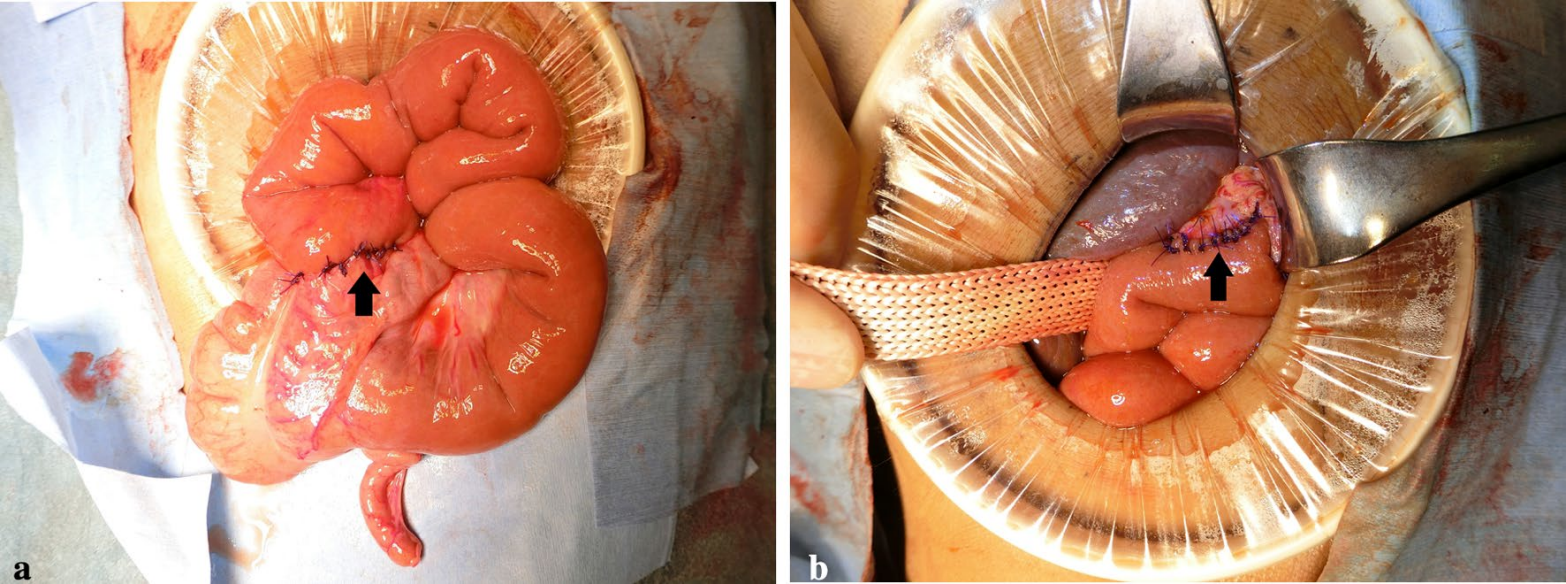
PIBD using a segment of jejunum (ante-colic) between the gall bladder and ascending colon. **A** Jejuno-colonic anastomosis (black arrow) **B** Cholecystojejunal anastomosis (black arrow). *PIBD* partial internal biliary diversion

The patient with FIC1 deficiency (case 1) rapidly developed liver failure at 11 years of age following infectious enteritis. Computed tomography scan of the abdomen showed a grossly distended gallbladder, and the patient underwent partial external biliary diversion (PEBD). He presented with refractory diarrhea, pancreatitis, and gradually worsening liver dysfunction. At 11.4 years of age, the patient underwent LDLT accompanied by total external biliary diversion (TEBD) using a segment of jejunum. The liver biopsy showed no evidence of steatohepatitis. However, the patient presented with refractory diarrhea, acute renal failure and pancreatitis, and developed the liver dysfunction due to repeated cellular rejection after LDLT. His condition gradually deteriorated due to uncontrolled septicemia and hemophagocytic syndrome. He eventually died of sepsis and other associated complications 6 months after LDLT (Fig. 1). The other patient with FIC1 deficiency (case 2) underwent partial internal biliary diversion (PIBD) at 9.8 years of age, owing to intractable pruritus. In PIBD, a 25 cm segment of jejunum was isolated and anastomosed between the termino-lateral side of the gall bladder and the mid-portion of the ascending colon. The continuity of the jejunum was established by jejuno-jejunal anastomosis (Fig. 2). Thereafter, pruritus largely resolved after PIBD.

#### BSEP deficiency: cases 3 and 4

The two patients with BSEP deficiency (Cases 3 and 4) presented with decompensated liver failure and underwent LDLT at 1.9 and 4.8 years of age, respectively. The clinical symptoms and laboratory findings of the first patient with BSEP deficiency (case 3) improved after LDLT. However, the patient developed symptoms of cholestatic liver disease at 6 months after LDLT. Immunofluorescence staining of normal human liver sections with the patient’s serum and the subsequent use of an anti–human IgG antibody to detect serum antibodies showed reactivity to the BSEP epitope in the canalicular membrane and revealed the deposition of antibodies against BSEP in the liver graft [26]. Treatment with plasmapheresis was initiated for the recurrence of BSEP deficiency, followed by the administration of intravenous immunoglobulin and rituximab. However, cholestasis and pruritus rapidly reoccurred after treatment. While waiting for a second transplantation, the patient’s condition deteriorated due to septicemia and necrotizing pancreatitis. He eventually died of gastrointestinal bleeding at 3 years and 4 months after LDLT. The second patient with BSEP deficiency (case 4) recovered well after LDLT and was discharged from the hospital. There was no evidence of posttransplant recurrence of cholestasis. Seventeen years after LDLT, the latest clinical findings showed normal liver function, and liver biopsy revealed the absence of hepatic fibrosis.

#### MDR3 deficiency: case 5

In the patient with MDR3 deficiency (case 5), despite the absence of any triggering factors, the patient’s condition rapidly progressed to terminal liver failure at 6 years of age. The patient presented with acute cellular rejection and hepatic artery stenosis 1 month after LDLT and, therefore, received anticoagulant and steroid pulse therapy. After the treatments, the patient recovered and was discharged from the hospital. The latest clinical findings 8 months after LDLT showed normal liver function, and there were no associated complications.

#### TJP2 deficiency: case 6

The patient with TJP2 deficiency (case 6) was histologically diagnosed with BSEP deficiency at 1 year of age based on the absence of BSEP, though she exhibited no significant genetic abnormalities. Her liver function gradually worsened, and she underwent LDLT at 4 years of age. The patient presented with drug-induced pancreatitis and acute renal failure, the latter of which was treated with continuous hemodiafiltration. She recovered well and was discharged from the hospital. Repeat genetic testing after LDLT showed a previously undetected significant mutation in the *TJP2* gene, and the patient was, therefore, diagnosed with TJP2 deficiency. The latest clinical findings showed normal liver function 5 years after LDLT, and there were no associated complications.

## Discussion

Biliary diversion is the first-line surgical management strategy for FIC1 deficiency; however, once cirrhosis has developed, patients often have poor outcomes [22]. Hence, non-transplant surgical management, such as external or internal biliary diversion, should be considered in patients with PFIC who do not have cirrhosis. Surgical biliary diversion with internal or external drainage for patients with PFIC decreased bile acid levels in the enterohepatic circulation and improved the outcomes of LT [33]. Total biliary diversion during LT has been documented as a possible explanation for FIC1 deficiency [34]. Hence, we performed TEBD accompanied by LDLT in a patient with FIC1 deficiency (Case 1). However, LDLT accompanied by total biliary diversion was ineffective. Meanwhile, PIBD was beneficial for the relief of pruritus in another patient with FIC1 deficiency (Case 2), and it also eliminated the disadvantages of a permanent stoma. The FIC1 deficiency recipient (Case 1) developed the diarrhea exacerbation after LDLT. The main cause of refractory diarrhea is thought to be the expression of the FIC1 protein in various organs, and higher expression levels are found in the small intestine [6]. After LDLT, a liver graft excretes a normal amount of bile acids into the bile, whereas the intestine remains functionally impaired. Consequently, increased bile acids in the small intestine and colon may cause refractory diarrhea [8, 35, 36]. These findings suggest that combined liver and intestinal transplantation may be needed to prevent complications after LT. When managing FIC1 deficiency, clinicians should consider genetic findings as well as the timing and indications of biliary diversion and LT.

In 2009, Keitel et al. first reported the recurrence of cholestasis after LT [23]. Several studies have subsequently reported that an inhibitory antibody against BSEP most likely causes cholestatic disease [23–26]. The complete absence of BSEP expression in the native liver is responsible for the failure to develop tolerance to BSEP. In addition, after the first transplantation, severe perfusion injury and severe graft damage have been suggested to result in the release of large amounts of BSEP protein derived from the donor graft [23]. Acute rejection episodes were recognized in one patient with BSEP deficiency (Case 3), who developed the recurrence of cholestasis after LDLT. However, there was no evidence of posttransplant complications in the other patient with BSEP deficiency (Case 4). Posttransplant development of anti-BSEP antibodies may also be associated with severe graft damage after LT. We recommend monitoring for the development of BSEP autoantibodies in patients who undergo LDLT for BSEP deficiency. With regard to donor selection for LDLT, previous reports have shown that LDLT has outcomes similar to those of LT from deceased donors [37, 38]. In Japan, the number of patients who undergo cadaverous LT is extremely low. Patients who undergo LDLT from related donors are not at high risk of failure due to PFIC-related causes [21, 22].

MDR3 and TJP2 deficiency are extremely rare, and only a few studies have reported the outcomes of LT in pediatric patients with MDR3 deficiency [20, 27–31] and TJP2 deficiency [13, 39]. In our cases, the clinical outcomes were good in the patients with MDR3 and TJP2 deficiency who underwent LDLT. MDR3 deficiency develops relatively late, with cholestatic symptoms developing in late infancy to adolescence [11, 12]. Previous reports showed that severe *ABCB4* genotypes are generally associated with reduced liver expression levels of MDR3 and are more prevalent in children with MDR3 deficiency [28, 40]. Colombo et al. showed that in children with mutations in two *ABCB4* alleles, a situation that is genetically similar to the presence of a radical mutation, there was rapid regression to terminal liver failure [28]. In our study, the patient with MDR3 deficiency presented with a homozygous mutation in the *ABCB4* gene, and mutations in both alleles might have caused rapid liver failure in early childhood.

## Conclusion

Although LDLT was considered an effective treatment for PFIC, the clinical courses and outcomes after LDLT were still inadequate in patients with FIC1 and BSEP deficiency. LDLT accompanied by total biliary diversion may not be as effective for patients with FIC1 deficiency.

## Data Availability

The data sets analysed during the current study are available from the corresponding author on reasonable request.

## Abbreviations

*ATP8B1*: ATPase class 8B member 1
*ABCB11*: ATP binding cassette subfamily B member 11
*ABCB4*: ATP binding cassette subfamily B member 4
ALT: Alanine aminotransferase
BSEP: Bile salt export pump
FIC1: Familial intrahepatic cholestasis 1
LT: Liver transplantation
LDLT: Living-donor liver transplantation
MDR3: Multidrug resistance protein 3
PEBD: Partial external biliary diversion
PFIC: Progressive familial intrahepatic cholestasis
PIBD: Partial internal biliary diversion
TEBD: Total external biliary diversion
*TJP2*: Tight junction protein 2
